# Micro RNA Expression after Ingestion of Fucoidan; A Clinical Study

**DOI:** 10.3390/md18030143

**Published:** 2020-02-28

**Authors:** Nuri Gueven, Kevin J. Spring, Sandra Holmes, Kiran Ahuja, Raj Eri, Ah Young Park, J Helen Fitton

**Affiliations:** 1 Pharmacy, School of Medicine, University of Tasmania, Hobart, TAS 7001, Australia; S.E.Holmes@utas.edu.au; 2 Medical Oncology, Ingham Institute for Applied Medical Research, School of Medicine, Western Sydney University and SWS Clinical School, UNSW, Sydney, NSW 2170, Australia; K.Spring@westernsydney.edu.au; 3 School of Health Sciences, University of Tasmania, Newnham, TAS 7248, Australia; kiran.ahuja@utas.edu.au (K.A.); raj.eri@utas.edu.au (R.E.); 4 Marinova Pty Ltd., Cambridge, TAS 7170, Australia; ahyoung.park@marinova.com.au

**Keywords:** fucoidan, clinical, microRNA, anti-inflammatory, anti-cancer

## Abstract

Fucoidans are a class of fucose-rich sulfated polysaccharides derived from brown macroalgae that exert a range of biological activities in vitro and in vivo. To generate an unbiased assessment of pathways and processes affected by fucoidan, a placebo-controlled double-blind pilot study was performed in healthy volunteers. Blood samples were taken immediately before and 24 h after ingestion of a single dose of 1 g of *Undaria pinnatifida* fucoidan (UPF) or placebo. Levels of isolated miRNAs were analyzed using Taqman Open Array Human MicroRNA panels. Out of 754 miRNAs screened, UPF affected a total of 53 miRNAs. Pathway analysis using the TALOS data analysis tool predicted 29 different pathways and processes that were largely grouped into cell surface receptor signaling, cancer-related pathways, the majority of which were previously associated with fucoidans. However, this analysis also identified nine pathways and processes that have not been associated with fucoidans before. Overall, this study illustrates that even a single dose of fucoidans has the potential to affect the expression of genes related to fundamental cellular processes. Moreover, it confirms previous data that fucoidans influence immunity, cancer cells, inflammation, and neurological function.

## 1. Introduction

Fucoidans, a class of fucose-rich sulfated polysaccharides, are derived from brown macroalgae and are associated with a large range of biological activities [1,2]. As a naturally occurring part of edible seaweed, it is part of the normal diet in many countries and consequently some fucoidan extracts have obtained ‘generally recognized as safe’ (GRAS) status in the US and received ‘novel foods’ approval in the EU. Fucoidans have long been noted as a selectin blocking compound that inhibits cell–cell interactions. This ability to disrupt cell–cell interactions is likely, at least in part, responsible for the potent anti-inflammatory activity of different fucoidan preparations. Fucoidans can also inhibit the adhesion of proteins and organisms to non-biological surfaces, and may help to inhibit biofouling [3]. Previous data using a yeast deletion library illustrated that fucoidans can interact with more fundamental cellular pathways than previously anticipated [4]. Not only did the yeast study confirm that cell surface signaling pathways are affected by fucoidan, this study identified many additional fundamental intracellular pathways such as ribosome biogenesis, peroxisome biogenesis, DNA damage repair, cell cycle control and energy metabolism, just to name a few. These results suggested that we have only begun to understand the breadth of interactions that fucoidans can have with biological systems. 

Fucoidans and fucoidan-rich foods are widely consumed in different areas of the world, either unintentionally in the form of algae-containing food products or intentionally based on perceived, anecdotal, or experimental evidence of biological effects. However, most intentional consumers of fucoidan products aim to alleviate or prevent very specific health related issues. Together with uncontrolled access to these products, this prevents a broader, unbiased understanding of the different effects that fucoidans can elicit in human consumers, both in terms of therapeutic activities as well as unanticipated effects that might remain unreported. Therefore, the current pilot study set out to provide a first unbiased insight how one particular fucoidan preparation from *Undaria pinnatifida* (UPF) would affect the plasma microRNA (miRNA) composition in healthy individuals.

miRNAs are small non-coding RNA molecules that are evolutionally conserved and are known to affect gene expression by different mechanisms [5]. Therefore, miRNAs are currently widely studied as biomarkers for a large range of indications including cancer, cardiovascular disease, obesity, inflammation, osteoporosis, and neurodegenerative diseases [6]. Moreover, far from simple biomarkers for some miRNAs, a direct pathological function has been established, which provides the opportunity to address them as therapeutic targets [6]. Nutrients can affect the serum expression of microRNAs, either exogenous or endogenous [7]. Currently there is only scarce information published on the effects of fucoidan on miRNA expression. A single study implicated one specific miRNA in the anti-tumor activity of fucoidan [8]. In this study, *Sargassum hemiphyllum* fucoidan upregulated miRNA29b in human hepato-carcinoma cells, which was associated with a reduction in cell growth, colony formation, and invasiveness. The authors proposed that miRNA29b was partially responsible for this effect by suppressing its downstream target DNMT3B, which increased the metastasis suppressor MTSS1 and inhibited Epithelial to Mesenchymal Transition (EMT) [8]. In a second study, *Sargassum hemiphyllum* fucoidan reportedly downregulated miRNA-29c and upregulated miRNA-17-5p, which was also associated with a suppression of EMT in breast cancer cell lines in vitro, while cell survival was reduced by activation of the IP3K/Akt pathway [9]. However, how fucoidan in both studies increased miRNA-29 expression remains unexplained so far. Beyond these in vitro studies, no information is currently available on other fucoidan miRNA interactions. Therefore, the current pilot study provides some information, which circulating miRNAs are affected in the plasma of healthy volunteers by exposure to a single oral dose of UPF. Using pathway analysis of the UPF-induced miRNA changes, our results substantiate the anti-cancer effects of fucoidan and a range of other activities that have been associated with fucoidan previously. In addition, the present study also identified novel pathways not formerly associated with fucoidan.

## 2. Results

In total, 754 miRNAs were screened for this analysis. When human plasma miRNAs were compared between baseline (0 h) and 24 h post-treatment, a total of 63 miRNAs were found to be differentially expressed in the placebo-treated individuals (19 up-regulated, 44 down-regulated). In comparison, in the UPF-treated individuals, 53 miRNA were identified to be differentially regulated (15 up-regulated, 38 down-regulated) in plasma (Table 1). The miRNAs that were differentially expressed between the placebo and UPF groups were also assessed. For the significantly upregulated miRNAs, only one miRNA (hsa-miR-34b) was common to both the placebo and UPF groups, while for the significantly downregulated miRNAs, 5 (hsa-miR-369-3p, hsa-miR-500, hsa-miR-548a, hsa-miR-548d-5p, hsa-miR-886-3p) were common to both the placebo and UPF groups. The remaining miRNAs that were either upregulated or downregulated were unique to each of the groups. 

Using TALOS (.v2) software, several cellular pathways were identified that could likely be affected by these miRNAs (Table 2). For the plasma of placebo-treated individuals, 13 pathways were identified, while the analysis of the plasma miRNAs of UPF-treated individuals identified 39 potentially affected pathways. Since some pathways overlapped between the placebo- and UPF-treated samples, these were excluded to highlight the 31 pathways that were selectively associated with UPF-exposure (Table 3). While the pathway analysis predicted several pathways that have been associated with fucoidans before, it also predicted pathways and processes not formerly associated with fucoidans (Table 3). 

## 3. Discussion

All living organisms have acquired the ability to rapidly respond to environmental stimuli including food. These responses are typically associated by altered gene expression to adequately ensure homeostasis of a range of metabolic, hormonal and physiological functions. Over the last 10 years, the regulation of gene expression by micro RNAs (miRNAs) has gained significant attention and multiple miRNAs have been identified that can either be used as biomarkers of physiological functions or are causative for different states of disease [6]. For example, food, such as milk, can influence serum miRNA expression both by delivery of exogenous miRNAs and influencing endogenous miRNAs, as recently reviewed by Cui et al [7].

This study, for the first time, illustrates that a single dose of *Undaria pinnatifida* fucoidan (UPF) is able to affect miRNA composition in the plasma of healthy individuals, which suggests that fucoidan actively can alter gene expression in individuals that consume fucoidan. 

Out of 754 tested miRNAs, 53 were differentially regulated by UPF. The subsequent pathway analysis identified 31 pathways that are predicted to be selectively influenced by UPF. Since it is well described that fucoidans directly affect signaling pathways associated with cell surface receptors, it was reassuring to observe that major membrane receptor pathways for growth factors such as BDNF, EGFR/ErbB, and insulin receptor were predicted by the pathway analysis, as well as the associated downstream signaling components such as MAPK. This surface-activity of fucoidan is also reflected by the prediction of fucoidan effects on focal adhesion in the present study, which supports previous observations that low molecular weight fucoidan can attenuate aortic aneurism [15]. Additionally, research into fucoidan effects via MAPK cascade includes curative effects on leishmaniosis [18], affecting M2 type macrophages, inhibiting cancer growth by modulating immune responses [19], and reducing cerebral reperfusion injury [20]. Fucoidan interferes with the binding of cancer cells to extracellular matrix [34].

It is acknowledged that direct fucoidan-receptor interactions are different from affecting a signaling pathway by altering the associated gene expression. Nevertheless, in most reports, no direct interaction but rather an indirect modulation of signaling was reported. For example, fermented *Laminaria japonica*, which contains fucoidan, increased serum BDNF levels in elderly subjects over a six week period [10]. Whether this effect translated to increased BDNF-signaling in those individuals was not reported. In an animal model of depression, chronic delivery of (*Fucus vesiculosus*) fucoidan inhibited clinical signs, blocked the increase in tyrosine hydroxylase expression in the localized areas of the brain and inhibited the decrease in BDNF mRNA expression in the hippocampus [11]. Whilst correlated, it is not known at present whether these changes led to a restored BDNF signaling. 

This suggests that our data needs to be interpreted cautiously and does not necessarily represent direct fucoidan-receptor interactions. Most likely, the actual interactions are significantly more complex. This is illustrated by several related studies. While fucoidan was reported to restore insulin stimulated glucose-uptake in adipocytes in vitro [15], no fucoidan-effects on insulin or glucose control were observed in healthy overweight subjects [16] and in one clinical trial fucoidan even increased insulin resistance [17].

It was previously reported that *Sargassum hemiphyllum* fucoidan up-regulated miRNA29b and down-regulated miRNA29c in different cancer cell lines, which was proposed by the authors as the reason for the anti-cancer activity of this particular fucoidan [8,9]. Although the current study did not detect any selective modulation of any of these miRNAs by UPF, this was not unexpected given the different test systems (cancer cell lines versus healthy individuals) and different sources for fucoidan (*Sargassum hemiphyllum* versus *Undaria pinnatifida*). In this context it is also important to note that our prior research illustrates that cancer cells can respond very differently compared to non-immortalized cells. While UPF effectively induced DNA damage in colorectal cancer cells, it had no effect on non-immortalized primary human skin fibroblasts [4], which could also account for the different miRNA responses observed by the different studies. 

Van Weelden et al. recently reviewed the different cancer pathways that are affected by fucoidan [35] and a range of these pathways were also predicted by our analysis. Our results specifically identified prostate cancer-related pathways, which supports the previous studies by Boo [28] and Choo [29]. Both studies reported inhibitory effects of Undaria fucoidan on prostate cancer cells in vitro. Fucoidan was also reported to affect estrogen signaling, a mechanism that was proposed to induce cancer cell apoptosis [21]. Fucoidan was reported to suppress the growth pro-myeloid cancer cells [32], which supports the results of the present study that predicted effects of UPF on chronic and acute myeloid leukemias. Fucoidan has also been shown to affect the growth of glioma cells [30] and inhibit glioma cell-induced angiogenesis [33]. Fucoidans can act as both anti-angiogenic and pro-angiogenic agents, dependent mostly on their molecular weight [27], which was also identified by the present study.

In addition, our analysis identified the process of endocytosis as a potential target for fucoidan. While cellular uptake of fucoidan might rely on clathrin-dependent endocytosis [22], previous studies reported that fucoidan can inhibit endocytosis in HeLa cells [23]. Fucoidan suppressed Ca^2+^ -dependent endocytosis, potentially by inhibition of agonist-induced Ca^2+^ responses [23]. Whilst the present study identified endochondral ossification, fucoidan has been associated with bone cell differentiation [24], but not specifically with endochondral ossification, an interaction that remains to be verified.

Interestingly, our analysis also predicted fucoidan-regulated pathways that have not previously been reported in the literature such as circadian rhythm and long-term potentiation. If these interactions can be confirmed by future studies in more detail, it might identify novel unexpected applications of dietary fucoidan products. The results of the current study justify future trials that will assess detailed pharmacokinetic parameters, as well as dose–effect relationships, to provide a rational approach towards optimized fucoidan dosing.

Ultimately, the molecular mechanism by which miRNA levels are altered in healthy individuals are unknown at present and will require systematic studies in the future. It can be speculated that these effects could be mediated directly via systemic uptake of small concentrations of fucoidan, or perhaps by perturbations of the microbiome that could cause indirect systemic effects. 

## 4. Materials and Methods 

### 4.1. Materials

If not otherwise stated, all chemicals were obtained from Sigma-Aldrich (Castle Hill, NSW, Australia). *Undaria pinnatifida* fucoidan (UPF) was obtained from Marinova (Cambridge, TAS, Australia). This material was provided with a quoted fucoidan purity of 85.1% (dry weight). The calculation of fucoidan purity requires several inputs that are determined using spectrophotometric assays. The total carbohydrate content of a hydrolyzed sample was determined using the phenol-sulfuric method of Dubois [36], while the uronic acid content was determined by spectrophotometric analysis of the hydrolyzed compound in the presence of 3-phenylphenol, based on a method described by Filisetti-Cozzi and Carpita [37]. Sulfate content was analyzed spectrophotometrically using a BaSO4 precipitation method (BaCl2 in gelatin), based on the work of Dodgson [38], and found to be 24.6%. The molecular weight profile was determined via gel permeation chromatography using a size-exclusion column and reported relative to Dextran standards, with peak molecular weight found at 47.7 kDa.

### 4.2. Test compounds

Test compounds (Placebo and experimental) were hand-filled by a pharmacist into gelatin capsules (size 1, opaque, pink, *The Capsule Guy*, Adelaide SA, Australia) without excipients and individually weighed. Placebo consisted of 250 mg ± 3 mg/capsule of microcrystalline cellulose powder, while experimental contained 500 mg ± 3 mg/capsule of *Undaria pinnatafida* extract (Marinova, Cambridge, Australia). Capsules were stored in bottles marked “A” and “B” respectively before transported to the test site. Apart from the pharmacist, all other members of the study (investigators and test subjects) remained blinded to the treatment until the analysis of data had been finalized. 

### 4.3. Trial design

The study was a double-blind randomised placebo control study. All protocols and procedures were approved by the University’s Human Ethics committee (H0015183). Healthy males, 25–65 of years, with no health conditions were recruited by a university newsletter. This study was designed as a pilot study with low participant numbers. Therefore, to avoid hormone-induced fluctuations of miRNA levels that would have required a much larger number of participants, the inclusion criteria were restricted to males. Exclusion criteria also included regular endurance sport, frequent recreational drug use, impaired digestive function, including occasional or regular use of laxatives, infection, drug related diarrhea, or regular use of food supplements (vitamins, herbs, plant extracts, especially aloe vera, seaweed/extracts, mushroom, spirulina, and spirulina juice). All subjects were informed about potential risks, read, and signed informed consent. Subjects ingested two capsules (size 1) with 500 mg fucoidan extract/capsule or placebo. Blood samples were taken immediately before the study capsules were ingested and 24 h later. Participants were randomised by a person not directly involved in recruitment, data collection or data analysis and was performed in blocks of 2; stratification was performed based on age and body surface area. Blood samples were collected from 9 participants for placebo and 8 for fucoidan in EDTA-blood collection tubes (BD). Plasma was prepared by centrifugation and stored at −80 °C until further processing.

### 4.4. miRNA Quantification

**RNA Isolation:** Individual plasma samples were extracted using a Norgen total RNA purification kit (Cat #17200, Norgen Biotek Corp, ON Canada) according to the Manufacturer’s instructions. Briefly, 200 µL of plasma from baseline (0 h) and treatment (24 h) groups were extracted and eluted in 50 µL elution buffer and quantified using the Qubit RNA HS (high sensitivity) and microRNA assay kits (Life Technologies), aliquoted and stored at −80 °C.

**TaqMan OpenArray:** The expression levels of 754 miRNAs were profiled using the Taqman OpenArray Human MicroRNA panels (PN: 4470189; Life Technologies Forster City, CA, USA) on a QuantStudio 12K Flex instrument. For all experimental groups, 3 µL (~10 ng) of total RNA was used for reverse transcription (RT) reactions using MegaPlex RT Primers Human Pool Set v3.0 (PN: 4444745; Pool A v2.1 and Pool B v3.0) according to the manufactures application note (Optimised protocol with low sample input for profiling human microRNA using the OpenArray platform) using a BioRad c1000 Touch thermal cycler. No-template controls were also included. Pre-amplification of RT products was performed using 5 µL RT reaction combined with the matching Megaplex PreAmp Primer Pool A v2.1 or B v3.0 and amplified using the BioRad thermal cycler. The pre-amplified products were diluted 1:40 in 0.1x TE pH 8. For each experimental set 10 µL of the diluted products were combined to give a total of 40 µL pooled sample. For both Pool A and Pool B groups, 22.5 µL of the pooled products were combined with an equivalent volume of TaqMan OpenArray Real-Time Master Mix and aliquoted into a 96 well plate. Then, 5 µL from each well were then transferred into a 384 well plate for loading onto OpenArray plates using an AccuFill robotic system. The OpenArray plates were run on a QuantStudio 12K Flex instrument (Life Technologies) and the raw data files were imported and analysed using the DataAssist software (Life Technologies). Failed reactions were excluded from analysis and undetermined C_T_ values for samples sets determined to have good amplifications were assigned a threshold value of 40, defining low abundance or absence of miRNA expression. Global mean normalisation was used to calculate relative fold change for miRNA expression.

### 4.5. Pathway Analysis

Before unblinding the treatment groups, pathway analysis was performed using miTALOS online software (v2; http://mips.helmholtz-muenchen.de/mitalos/#/search) [39]. TALOS associates up- or down-regulated miRNAs with biological processes that are derived from three major pathway databases KEGG, WikiPathways and Reactome [39]. The software default settings for human samples and *Target Scan* as prediction tool were employed. Therefore, three miRNAs listed in Table 1 (rno-miR-29c#, mmu-let-7d#, and mmu-miR-374-5p) had to be excluded based on their nomenclature. No cell lines or tissues were pre-selected. A *p*-value of <0.05 was considered significant. The final data (Table 3) represents the differentially represented pathways in the UPF-treated cohort compared to the placebo-treated cohort. Pathways identified in both treatments were excluded from Table 3. 

## 5. Conclusions

The modulation of serum miRNA expression after acute administration of a single dose of Undaria-derived fucoidan indicates potential activity in several biological pathways, some of which have not previously been identified. 

## Figures and Tables

**Table 1:** Significantly up- or down-regulated miRNAs (basal vs. 24 h post treatment).

Placebo				UPF			
Up-regulated		Down-regulated		Up-regulated		Down-regulated	
Name	fold *	Name	fold *	Name	fold *	Name	fold *
hsa-miR-584	14.068	hsa-miR-193b#	13.219	hsa-miR-1247	13.767	hsa-miR-183#	17.262
hsa-miR-9	13.72	hsa-miR-500	13.17	hsa-miR-200b	12.888	hsa-miR-107	16.182
hsa-miR-656	13.528	hsa-miR-199a	13.12	hsa-miR-1180	12.714	hsa-miR-339-5p	15.277
hsa-miR-485-3p	12.983	hsa-miR-326	12.827	hsa-miR-623	11.927	hsa-miR-335#	13.787
hsa-miR-34b	11.862	hsa-miR-502	12.712	hsa-miR-135a	11.879	hsa-miR-299-5p	12.97
hsa-miR-605	11.565	hsa-miR-381	12.058	hsa-miR-1303	11.094	hsa-miR-199b	12.227
rno-miR-29c#	10.092	hsa-miR-213	11.713	hsa-miR-380-5p	9.369	hsa-miR-369-3p	11.453
hsa-miR-99a#	10.085	hsa-miR-369-3p	11.527	hsa-miR-518e	7.745	hsa-miR-29b-2#	11.401
hsa-miR-361-3p	10.017	hsa-miR-1	11.215	hsa-miR-454#	6.642	hsa-miR-27a#	11.007
hsa-miR-30d#	9.587	hsa-miR-886-3p	11.196	hsa-miR-34b	3.802	hsa-miR-520h	10.762
hsa-miR-196b	8.419	hsa-miR-424	10.948	hsa-miR-638	3.667	hsa-miR-500	10.741
hsa-miR-651	7.135	hsa-miR-363#	10.611	hsa-miR-517c	3.016	hsa-miR-9#	10.691
hsa-miR-18a#	5.996	hsa-miR-301b	10.506	hsa-miR-502-3p	2.934	hsa-miR-886-3p	10.258
hsa-miR-9#	5.242	hsa-miR-337-5p	10.262	hsa-miR-662	2.427	hsa-miR-487a	10.243
hsa-miR-452	5.228	hsa-miR-362-3p	10.232	hsa-miR-512-3p	2.411	hsa-miR-20a#	9.263
hsa-miR-324-3p	5.154	hsa-miR-184	10.091			hsa-miR-542-5p	9.079
hsa-miR-365	3.769	hsa-miR-876-5p	10.011			hsa-miR-548a	7.654
hsa-miR-454	3.625	hsa-miR-376b	9.925			hsa-miR-548d-5p	7.262
hsa-miR-494	2.905	hsa-miR-548d-5p	9.678			hsa-miR-520f	6.821
		hsa-miR-450a	9.491			hsa-miR-486-3p	5.978
		hsa-miR-362	8.547			hsa-miR-590-3P	3.606
		hsa-miR-1285	8.325			hsa-miR-296	3.308
		hsa-miR-545#	8.313			hsa-miR-509-5p	3.284
		hsa-miR-517c	8.044			hsa-miR-125a-5p	3.194
		hsa-miR-517b	7.977			hsa-miR-28-3p	3.083
		hsa-miR-454#	7.972			hsa-miR-342-5p	3.071
		hsa-miR-214#	7.444			hsa-miR-489	2.897
		hsa-miR-519e#	7.373			mmu-let-7d#	2.741
		hsa-miR-579	3.677			hsa-miR-34a	2.505
		hsa-miR-135a	3.402			hsa-miR-203	2.487
		hsa-miR-501-3p	3.177			mmu-miR-374-5p	2.410
		hsa-miR-29c	3.123			hsa-miR-652	2.388
		hsa-miR-218	2.999			hsa-miR-339-3p	2.197
		hsa-miR-512-3p	2.908			hsa-miR-1249	2.193
		hsa-miR-520e	2.54			hsa-miR-31	2.174
		hsa-miR-1180	2.488			hsa-miR-551b	2.114
		hsa-miR-522	2.46			hsa-miR-145#	2.029
		hsa-miR-133a	2.456			hsa-miR-411	2.024
		hsa-miR-548a	2.439				
		hsa-miR-425#	2.438				
		hsa-miR-664	2.403				
		hsa-miR-93#	2.173				
		mmu-miR-491	2.145				
		hsa-miR-625#	2.074				

* Only fold changes >2 were considered in the analysis.

**Table 2:** Predicted pathways (basal vs. 24 h post treatment).

**Placebo**			
**Source**	**Name**	***E***	***p*** **-Value**
kegg	Signaling pathways regulating pluripotency of stem cells	1.770	9.1 × 10^−4^
wp	Nuclear Receptors	2.871	1.5 × 10^−3^
kegg	Axon guidance	1.761	1.9 × 10^−3^
wp	TGF Beta Signaling Pathway	2.331	2.1 × 10^−3^
wp	Mesodermal Commitment Pathway	1.657	2.4 × 10^−3^
wp	BMP Signalling and Regulation	6.252	4.5 × 10^−3^
wp	Leptin signaling pathway	1.875	9.1 × 10^−3^
wp	Endoderm Differentiation	1.576	9.4 × 10^−3^
kegg	Thyroid cancer	2.566	1.6 × 10^−2^
kegg	Pancreatic cancer	1.849	1.7 × 10^−2^
wp	Wnt Signaling Pathway and Pluripotency	1.613	2.2 × 10^−2^
wp	Serotonin Receptor 4-6-7 and NR3C Signaling	2.865	3.4 × 10^−2^
kegg	Non-small cell lung cancer	1.808	3.6 × 10^−2^
**UPF-treated**			
**Source**	**Name**	***E***	***p*** **-Value**
wp	BDNF signaling pathway	2.481	1.0 × 10^−5^
wp	EGF-EGFR Signaling Pathway	2.271	1.6 × 10^−5^
kegg	Axon guidance	2.208	2.2 × 10^−4^
kegg	ErbB signaling pathway	2.568	4.7 × 10^−4^
kegg	Endocytosis	1.671	1.1 × 10^−3^
wp	Insulin Signaling	1.812	1.2 × 10^−3^
kegg	Signaling pathways regulating pluripotency of stem cells	1.837	1.9 × 10^−3^
wp	Endochondral Ossification	2.699	2.0 × 10^−3^
kegg	Focal adhesion	1.605	2.8 × 10^−3^
kegg	Prostate cancer	2.150	3.0 × 10^−3^
wp	ErbB Signaling Pathway	2.801	3.3 × 10^−3^
wp	TSH signaling pathway	2.491	3.6 × 10^−3^
kegg	Renal cell carcinoma	2.312	6.3 × 10^−3^
wp	TGF beta Signaling Pathway	1.728	6.8 × 10^−3^
kegg	Glioma	2.267	7.9 × 10^−3^
kegg	Rap1 signaling pathway	1.500	9.1 × 10^−3^
wp	Leptin signaling pathway	2.087	9.4 × 10^−3^
kegg	Adherens junction	2.050	1.2 × 10^−2^
wp	MAPK Cascade	3.885	1.2 × 10^−2^
kegg	Chronic myeloid leukemia	2.050	1.2 × 10^−2^
wp	Wnt Signaling Pathway and Pluripotency	1.797	1.2 × 10^−2^
wp	Regulation of Microtubule Cytoskeleton	2.557	1.4 × 10^−2^
kegg	Proteoglycans in cancer	1.462	1.6 × 10^−2^
wp	Mesodermal Commitment Pathway	1.558	1.6 × 10^−2^
wp	Oncostatin M Signaling Pathway	2.074	1.6 × 10^−2^
wp	Signaling Pathways in Glioblastoma	1.868	1.6 × 10^−2^
wp	Angiogenesis	5.592	1.8 × 10^−2^
kegg	Circadian rhythm	3.232	1.9 × 10^−2^
kegg	Neurotrophin signaling pathway	1.642	1.9 × 10^−2^
kegg	FoxO signaling pathway	1.574	2.2 × 10^−2^
kegg	Acute myeloid leukemia	2.105	2.3 × 10^−2^
kegg	Pathways in cancer	1.273	2.9 × 10^−2^
kegg	TGF-beta signaling pathway	1.748	3.5 × 10^−2^
wp	Androgen receptor signaling pathway	1.685	3.6 × 10^−2^
kegg	Hippo signaling pathway	1.462	3.8 × 10^−2^
kegg	Long-term potentiation	1.829	4.0 × 10^−2^
kegg	Estrogen signaling pathway	1.600	4.3 × 10^−2^
wp	Physiological and Pathological Hypertrophy of the Heart	3.106	4.9 × 10^−2^
wp	Wnt Signaling Pathway	1.793	4.9 × 10^−2^

**Table 3:** Predicted pathways selective for UPF-treatment (basal vs. 24 h post treatment).

Pathways Affected by UPF-treatment				
Source	Signaling Pathways	Prior Evidence	References	*p*-Value
wp	BDNF signaling pathway	Yes	Reid and Lee [10,11]	1.0 × 10^−5^
wp	EGF-EGFR Signaling Pathway	Yes	Wang [12]	1.6 × 10^−4^
kegg	ErbB signaling pathway	Yes	Thakur [13]	4.7 × 10^−4^
wp	Insulin Signaling	Yes	Wang, Sim [14,15], Wright [16], Hernadez [17]	1.2 × 10^−3^
wp	TSH signaling pathway	No	-	3.6 × 10^−3^
kegg	Rap1 signaling pathway	No	-	9.1 × 10^−3^
wp	MAPK Cascade	Yes	Sharma [18] Sun [19] Che [20]	1.2 × 10^−2^
wp	Oncostatin M Signaling Pathway	No	-	1.6 × 10^−2^
kegg	Neurotrophin signaling pathway	No	-	1.9 × 10^−2^
kegg	FoxO signaling pathway	No	-	2.2 × 10^−2^
kegg	Hippo signaling pathway	No	-	3.8 × 10^−2^
kegg	Estrogen signaling pathway	Yes	Zhang [21]	4.3 × 10^−2^
	**Cellular Processes**			
kegg	Endocytosis	Yes	Zhang [22], Wu [23]	1.1 × 10^−3^
wp	Endochondral Ossification	Yes	Carson [24]	2.0 × 10^−3^
kegg	Focal adhesion	Yes	Zhou [25]	2.8 × 10^−3^
kegg	Adherens junction	No	-	1.2 × 10^−2^
wp	Regulation of Microtubule Cytoskeleton	Yes	Park (query) [26]	1.4 × 10^−2^
wp	Angiogenesis	Yes	Ustyuzhanina [27]	1.8 × 10^−2^
kegg	Circadian rhythm	No	-	1.9 × 10^−2^
kegg	Long-term potentiation	No	-	4.0 × 10^−2^
	**Cancer related pathway**			
kegg	Prostate cancer	Yes	Boo [28] and Choo [29]	3.0 × 10^−3^
kegg	Renal cell carcinoma	No	-	6.3 × 10^−3^
kegg	Glioma	Yes	Ko [30]	7.9 × 10^−3^
kegg	Chronic myeloid leukemia	Yes	Jin [31], Astashrazm [32]	1.2 × 10^−2^
wp	Signaling Pathways in Glioblastoma	Yes	Ko [30], lv [33]	1.6 × 10^−2^
kegg	Proteoglycans in cancer	Yes	Liu [34]	1.6 × 10^−2^
kegg	Acute myeloid leukemia	Yes	Jin [31], Astashrazm [32]	2.3 × 10^−2^
kegg	Pathways in cancer	Yes	Corban [4], Van Weelden [35]	2.9 × 10^−2^
	**Other disease states**			
wp	Physiological and Pathological Hypertrophy of the Heart	No	-	4.9 × 10^−2^

## References

[1] Fitton H.J., Stringer D.S., Park A.Y., Karpiniec S.N. (2019). Therapies from Fucoidan: New Developments. Mar. Drugs.

[2] Fitton J.H., Stringer D.N., Karpiniec S.S., Park A.Y. (2019). The manufacture, characterization, and uses of fucoidans from macroalgae. Enzymatic Technologies for Marine Polysaccharides.

[3] Jeong Y., Thuy L.T., Ki S.H., Ko S., Kim S., Cho W.K., Choi J.S., Kang S.M. (2018). Multipurpose Antifouling Coating of Solid Surfaces with the Marine-Derived Polymer Fucoidan. Macromol. Biosci..

[4] Corban M., Ambrose M., Pagnon J., Stringer D., Karpiniec S., Park A., Eri R., Fitton J.H., Gueven N. (2019). Pathway Analysis of Fucoidan Activity Using a Yeast Gene Deletion Library Screen. Mar. Drugs.

[5] Bartel D.P. (2009). MicroRNAs: Target recognition and regulatory functions. Cell.

[6] Mori M.A., Ludwig R.G., Garcia-Martin R., Brandao B.B., Kahn C.R. (2019). Extracellular miRNAs: From Biomarkers to Mediators of Physiology and Disease. Cell Metab..

[7] Cui J., Zhou B., Ross S.A., Zempleni J. (2017). Nutrition, microRNAs, and Human Health. Adv. Nutr..

[8] Yan M.D., Yao C.J., Chow J.M., Chang C.L., Hwang P.A., Chuang S.E., Whang-Peng J., Lai G.M. (2015). Fucoidan Elevates MicroRNA-29b to Regulate DNMT3B-MTSS1 Axis and Inhibit EMT in Human Hepatocellular Carcinoma Cells. Mar. Drugs.

[9] Wu S.Y., Wu A.T., Yuan K.S., Liu S.H. (2016). Brown Seaweed Fucoidan Inhibits Cancer Progression by Dual Regulation of mir-29c/ADAM12 and miR-17-5p/PTEN Axes in Human Breast Cancer Cells. J. Cancer.

[10] Reid S.N.S., Ryu J.K., Kim Y., Jeon B.H. (2018). The Effects of Fermented Laminaria japonica on Short-Term Working Memory and Physical Fitness in the Elderly. Evid. Complement. Altern. Med..

[11] Lee B., Shim I., Lee H., Hahm D.H. (2013). Fucoidan prevents depression-like behavior in rats exposed to repeated restraint stress. J. Nat. Med..

[12] Wang W., Wu J., Zhang X., Hao C., Zhao X., Jiao G., Shan X., Tai W., Yu G. (2017). Inhibition of Influenza A Virus Infection by Fucoidan Targeting Viral Neuraminidase and Cellular EGFR Pathway. Sci. Rep..

[13] Thakur V., Lu J., Roscilli G., Aurisicchio L., Cappelletti M., Pavoni E., White W.L., Bedogni B. (2017). The natural compound fucoidan from New Zealand Undaria pinnatifida synergizes with the ERBB inhibitor lapatinib enhancing melanoma growth inhibition. Oncotarget.

[14] Wang J., Liu H., Li N., Zhang Q., Zhang H. (2014). The protective effect of fucoidan in rats with streptozotocin-induced diabetic nephropathy. Mar. Drugs.

[15] Sim S.Y., Shin Y.E., Kim H.K. (2019). Fucoidan from Undaria pinnatifida has anti-diabetic effects by stimulation of glucose uptake and reduction of basal lipolysis in 3T3-L1 adipocytes. Nutr. Res..

[16] Wright C.M., Bezabhe W., Fitton J.H., Stringer D.N., Bereznicki L.R.E., Peterson G.M. (2019). Effect of a Fucoidan Extract on Insulin Resistance and Cardiometabolic Markers in Obese, Nondiabetic Subjects: A Randomized, Controlled Trial. J. Altern. Complement. Med..

[17] Hernandez-Corona D.M., Martinez-Abundis E., Gonzalez-Ortiz M. (2014). Effect of fucoidan administration on insulin secretion and insulin resistance in overweight or obese adults. J. Med. Food.

[18] Sharma G., Kar S., Basu Ball W., Ghosh K., Das P.K. (2014). The curative effect of fucoidan on visceral leishmaniasis is mediated by activation of MAP kinases through specific protein kinase C isoforms. Cell. Mol. Immunol..

[19] Sun J., Sun J., Song B., Zhang L., Shao Q., Liu Y., Yuan D., Zhang Y., Qu X. (2016). Fucoidan inhibits CCL22 production through NF-kappaB pathway in M2 macrophages: A potential therapeutic strategy for cancer. Sci. Rep..

[20] Che N., Ma Y., Xin Y. (2017). Protective Role of Fucoidan in Cerebral Ischemia-Reperfusion Injury through Inhibition of MAPK Signaling Pathway. Biomol. Ther. (Seoul).

[21] Zhang J., Riby J.E., Conde L., Grizzle W.E., Cui X., Skibola C.R. (2016). A Fucus vesiculosus extract inhibits estrogen receptor activation and induces cell death in female cancer cell lines. BMC Complement. Altern. Med..

[22] Zhang E., Chu F., Xu L., Liang H., Song S., Ji A. (2018). Use of fluorescein isothiocyanate isomer I to study the mechanism of intestinal absorption of fucoidan sulfate in vivo and in vitro. Biopharm. Drug Dispos..

[23] Wu H., Gao S.-B., Sakurai T., Terakawa S. (2011). Fucoidan suppresses endocytosis in cultured HeLa cells. Chin. J. Integr. Med..

[24] Carson M.A., Clarke S.A. (2018). Bioactive Compounds from Marine Organisms: Potential for Bone Growth and Healing. Mar. Drugs.

[25] Zhou M., Ding Y., Cai L., Wang Y., Lin C., Shi Z. (2018). Low molecular weight fucoidan attenuates experimental abdominal aortic aneurysm through interfering the leukocyte-endothelial cells interaction. Mol. Med. Rep..

[26] Park H.S., Kim G.Y., Nam T.J., Deuk Kim N., Hyun Choi Y. (2011). Antiproliferative activity of fucoidan was associated with the induction of apoptosis and autophagy in AGS human gastric cancer cells. J. Food Sci..

[27] Ustyuzhanina N.E., Bilan M.I., Ushakova N.A., Usov A.I., Kiselevskiy M.V., Nifantiev N.E. (2014). Fucoidans: Pro- or antiangiogenic agents?. Glycobiology.

[28] Boo H.J., Hong J.Y., Kim S.C., Kang J.I., Kim M.K., Kim E.J., Hyun J.W., Koh Y.S., Yoo E.S., Kwon J.M. (2013). The anticancer effect of fucoidan in PC-3 prostate cancer cells. Mar. Drugs.

[29] Choo G.S., Lee H.N., Shin S.A., Kim H.J., Jung J.Y. (2016). Anticancer Effect of Fucoidan on DU-145 Prostate Cancer Cells through Inhibition of PI3K/Akt and MAPK Pathway Expression. Mar. Drugs.

[30] Do H., Pyo S., Sohn E.H. (2009). Suppression of iNOS expression by fucoidan is mediated by regulation of p38 MAPK, JAK/STAT, AP-1 and IRF-1, and depends on up-regulation of scavenger receptor B1 expression in TNF-alpha- and IFN-gamma-stimulated C6 glioma cells. J. Nutr. Biochem..

[31] Jin J.O., Song M.G., Kim Y.N., Park J.I., Kwak J.Y. (2010). The mechanism of fucoidan-induced apoptosis in leukemic cells: Involvement of ERK1/2, JNK, glutathione, and nitric oxide. Mol. Carcinog..

[32] Atashrazm F., Lowenthal R.M., Woods G.M., Holloway A.F., Karpiniec S.S., Dickinson J.L. (2016). Fucoidan Suppresses the Growth of Human Acute Promyelocytic Leukemia Cells In Vitro and In Vivo. J. Cell. Physiol..

[33] Lv Y., Song Q., Shao Q., Gao W., Mao H., Lou H., Qu X., Li X. (2012). Comparison of the effects of marchantin C and fucoidan on sFlt-1 and angiogenesis in glioma microenvironment. J. Pharm. Pharmacol..

[34] Liu J.M., Bignon J., Haroun-Bouhedja F., Bittoun P., Vassy J., Fermandjian S., Wdzieczak-Bakala J., Boisson-Vidal C. (2005). Inhibitory effect of fucoidan on the adhesion of adenocarcinoma cells to fibronectin. Anticancer Res..

[35] Van Weelden G., Bobiński M., Okła K., Van Weelden W.J., Romano A., Pijnenborg J.M.A. (2019). Fucoidan Structure and Activity in Relation to Anti-Cancer Mechanisms. Mar. Drugs.

[36] DuBois M., Gilles K.A., Hamilton J.K., Rebers P.A., Smith F. (1956). Colorimetric Method for Determination of Sugars and Related Substances. Anal. Chem..

[37] Filisetti-Cozzi T.M.C.C., Carpita N.C. (1991). Measurement of uronic acids without interference from neutral sugars. Anal. Biochem..

[38] Dogson K.S. (1961). Determination of inorganic sulphate in studies on the enzymic and non-enzymic hydrolysis of carbohydrate and other sulphate esters. Biochem. J..

[39] Preusse M., Theis F.J., Mueller N.S. (2016). miTALOS v2: Analyzing Tissue Specific microRNA Function. PLoS ONE.

